# Pelt Biting as a Practical Indicator of Social and Environment Stress in Farmed Red Deer

**DOI:** 10.3390/ani11113134

**Published:** 2021-11-02

**Authors:** Francisco Javier Pérez-Barbería, Andrés José García, María López-Quintanilla, Tomás Landete-Castillejos

**Affiliations:** Game and Livestock Resources Unit, University of Castilla-La Mancha, IDR, IREC, 02071 Albacete, Spain; AndresJose.Garcia@uclm.es (A.J.G.); Maria.LQuintanilla@uclm.es (M.L.-Q.); Tomas.Landete@uclm.es (T.L.-C.)

**Keywords:** agonistic interactions, animal welfare indicator, stress, red deer, management

## Abstract

**Simple Summary:**

Agonistic behavioural interactions play a decisive role in the competition for food, space, mating opportunities, and establishing social rank. We propose the use of the number of bites on the pelt of red deer as an index of agonistic interactions between group members. Using behavioural data from a 14-year time series of a captive population of Iberian red deer (*Cervus elaphus*), we found that deer that were of higher social rank, heavier, living in smaller groups, or under no heat stress conditions suffer less pelt bites than those of lower social rank, lighter, living in bigger groups, or under heat stressing conditions. Hinds that gave birth earlier in the parturition period suffered less pelt biting than those that gave birth around the peak of the parturition season. Pelt biting is useful to identify management situations in which deer welfare could be at stake.

**Abstract:**

Agonistic behavioural interactions play a decisive role in the competition for food, space, mating opportunities, and establishing social rank. We used pelt biting (number of bites on an animal’s body) as a proxy for assessing the intensity of agonistic animal interactions and how it responded to social, population, and heat stress factors. We modelled a 14-year time series of pelt biting records and observational data of agonistic interactions on a population of captive Iberian red deer (*Cervus elaphus*). We found that (i) the higher the social rank of deer, the lower the number of pelt bites received; (ii) increasing heat stress conditions caused deer to suffer more pelt bites; (iii) males received more bites than females; (iv) the heavier the deer, the lower the number of bites on their bodies; (v) the bigger the group, the more bites exhibited on its members; (vi) deer 5–6 years old suffered greater rate of pelt biting than younger or older deer; and (vii) hinds that gave birth earlier in the parturition period suffered less pelt biting than those that gave birth around the peak of the parturition season (*p* < 0.01 for all effects). Pelt biting is useful to predict management situations in which deer welfare could be at stake.

## 1. Introduction

Animal societies are driven by a complex network of interactions between group members, and one type of interaction facilitates the establishment of the social rank between individuals, such as agonistic interactions [1]. Social rank and group structure are the expression of the dominant/subordinate role of the group members, which reflects the individual skills required to gain access to limited resources, for example, space, food, and mating opportunities [2,3,4]. Social rank within a group is dynamic [1,5,6], as it is affected not only by the outcome of continuous social interactions but also environmental factors that impact on resource availability [7,8]. Group size affects the intensity of agonistic interactions and the type and stability of the social structure. Small groups tend to have linear ranks and are more stable than bigger groups [9], since aggressiveness increases with group size, because the availability of resources per individual declines [10]. Individual traits, such as sex, size, and age, are instrumental in the outcome of social interactions and group structure [11,12].

External abiotic stressors, such as heat [13], which affect aggregation patterns, comfort, or hormone levels, can also determine aggression activity [10,14]. Global climate models predict an increase in air temperature in the next few decades [15], and heat stress has been demonstrated to be an important stressor in domestic ruminants and in farmed deer [13,16]. It is recognised that good animal welfare and optimal levels of production are closely related, but there are benefits of improving animal welfare that extend beyond production gains and minimum legal requirements [17]. Animal aggression in farms is one of the main factors affecting livestock welfare, and it has direct repercussions on the economic viability of the exploitation [18].

Ungulate species display a repertoire of agonistic behavioural interactions to establish their social rank. These behaviours range from vocalisations, displays of dominance or submission, butting, biting, kicking, chasing, and changes in the distance between individuals, and some can escalate to aggressive behaviours that can lead to injuries and even death [6,19,20,21,22]. Recording animal interactions in large groups is a time consuming task [23]. In the last decade, automatic devices have eased the collection of data for monitoring animal behaviour, specially spatial movement, from which social interactions can be inferred [21,24]. However, these devices are expensive and require complex logistics for their deployment.

In red deer, one aggressive behaviour is biting the pelt of their peers [20,25]. The result of this behaviour can be observed on the animal´s pelt as a number of bite-size patches of fur that have been plucked. We assessed whether pelt biting can be efficiently used as a practical indicator to record agonistic interactions in captive red deer and how this behaviour is modulated by sex, body size, age, social rank, population density, and environmental stressors. We also tested some social predictions on animal interactions and discussed the potential applications of pelt biting as a useful indicator of social and environmental distress in deer farms.

## 2. Materials and Methods

### 2.1. Hypotheses

Two groups of hypotheses were tested on the response of pelt biting, related to (i) the hierarchical rank of the deer and related body traits, which correlate positively with successful outcome in competition for resources, and (ii) the abiotic environmental stress where they live (Table 1). It is hypothesised that males individuals (H4) [26], younger individuals (H3), lower hierarchical rank individuals (H1), smaller body size (i.e., body weight, H2) individuals, and those exposed to heat stress (H6) suffer higher rates of pelt biting (Table 1).

Sackett et al. [27], in a colony of captive pigtail monkeys (*Macaca menestrina*), found that mothers carrying female foetuses were more frequently harassed and bitten than those carrying male foetuses (H5, Table 1). They hypothesised that this was related to mother´s hierarchical status, contingent upon foetus sex-related hormone levels. Namely, during the second half of pregnancy, foetal male gonads begin secreting testosterone and producing a sharp increase in transplacental maternal circulating testosterone [28], which might induce maternal behavioural changes and produce chemicals that are exudate, making it possible for peers to smell them and so identify their condition.

### 2.2. Pelt Biting

**Figure 1:**
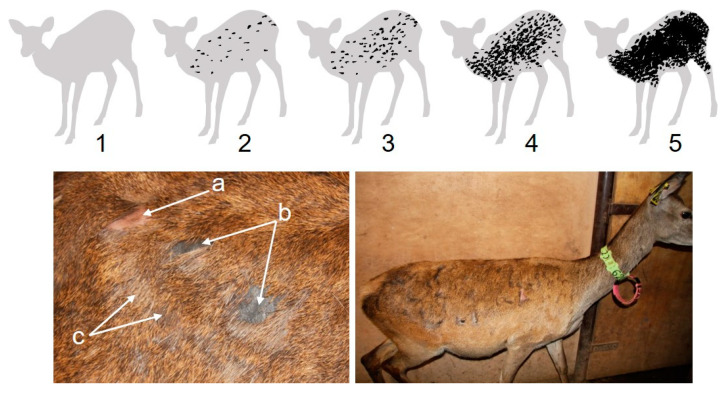
Pelt biting on red deer. Top: pelt biting class 1 to class 5 (see description in section Pelt biting). Light grey is intact pelt; black represents pelt areas that have been bitten. Bottom left: three types of bites on pelt, a. pelt biting has exposed the skin, which is light pink when the bite is recent, hair has been plucked from the root; b. old pelt bites, hairless skin area has turned grey in colour; c. re-grown hair. Bottom right: hind showing pelt biting class 1.75.

In our farmed red deer, we observed body biting between conspecific deer as a direct consequence of agonistic behaviour. Deer bit their peers in four main situations: when (i) deer were spatially very close together, (ii) competing for food at feed bins, (iii) a deer actively approached another and bit it, and more rarely, (iv) as a retaliation to an aggression. In general, biting behaviour was performed as a single bite, and the bitten deer reacted immediately by moving a few steps away, whereupon no more interactions took place. Normally, a bite produced a conspicuous hairless patch on the pelt of the bitten animal, as a consequence of hair removal by plucking action (i.e., hair removal from the root). In some cases, when the biting strength was light, no hair was apparently plucked. Due to these observations, we undoubtedly discard the theory that hairless patches on the pelt of our deer were a consequence of self- or allo-grooming, ecto-parasites, mycosis, or mineral deficiency. A single bite produces a conspicuous bald area, no larger than 5 cm^2^; multiple bites can overlap and produce larger bitten surfaces on the deer pelt. Just after biting, the exposed skin is pink-pale in colour and, after a few days, turns grey. The bald area can be easily identified even when hair is re-growing, as the new hair is lighter in colour in comparison with the surrounding hair (Figure 1).

Pelt biting monitoring was performed in each deer once a week by visual inspection of the right side of the deer when they were handled for routine health, body weight, and condition assessment. The reason behind assessing pelt biting on the right side of the animal was for practical convenience, as this was the side visually exposed when the animal was in the handling pen. Pelt biting was carried out by the same observer (AJG) across the duration of the study. We categorised the extension of pelt biting into five ordinal main classes as follows (Figure 1). Class 1, no bites to very few bites, [0–3%] of pelt surface bitten (pelt surface is defined as the body surface with the exception of head, backstrap, lower parts of limbs, and lower parts of abdomen and genital area); class 2, few bites on shoulder, flank, lower rump, and upper and middle parts of the haunch, (3–10%] of pelt surface bitten; class 3, frequent bites on body parts described in class 2, bites start to appear on the neck, (10–40%] of the pelt surface bitten; class 4, abundant biting on body parts of classes 2 and 3, bites start to overlap, producing continuous bald areas, bites start to appear on the shanks of fore and rear limbs, (40–70%] of the pelt surface bitten; class 5, great extension of the surface of the pelt bitten, bites overlapping, abundant bites on shanks, >70% of the pelt surface bitten. These five main classes were further divided into ordinal quartiles categories (e.g., 2.25 = class 2 plus 25% of the area comprised between class 2 and class 3). The percentage of pelt surface bitten to produce this classification was calculated using ImageJ software [29] applied to red deer pictures taken on lateral view and representative of each of the pelt biting classes.

### 2.3. Data and Animals

Data collection was carried out at the University of Castilla-La Mancha (UCLM) deer farm experimental facilities (38°57′32.8′′ N 1°52′51.8′′ W, Albacete, Spain) between 2006 and 2019. The climate was continental Mediterranean, cooler summers and greater variation in seasonal temperatures than the typical Mediterranean climate, bordering a cold semi-arid climate (annual mean min and max temperature = 5.9 °C December and 24.3 °C July; min–max rainfall = 12 mm July, 42 mm October; http://crea.uclm.es/siar/datmeteo/ (accessed on 21 April 2021). The study used 427 red deer females and 424 males (Table 2).

Females’ age ranged between 1 and 21 years old (mean = 4.7, Q1 = 1.1, Q3 = 7.1) and males’ age between 1 and 15 (mean = 1.7, Q1 = 0.4, Q3 = 2.2). Mean body weight in females was 85.7 kg (Q1 = 71, Q3 = 105) and 93 kg in males (Q1 = 48, Q3 = 129). Females and males were split into 2–6 groups, depending on the number of deer in the farm; each group was allocated to different fields of size between 0.6 and 1.2 ha (mean density in fields = 25 deer/ha). Deer relied entirely on supplementary feed, as the amount of grass provided by the fields was negligible. The base diet year-round was a well-balanced mixture of chopped alfalfa hay and orange pulp, supplied ad libitum three times a week, and between March and October, this was supplemented with pelleted feed. Feed was presented to deer on both-side access 14 m long belt feeders to minimise aggressions during feeding [13]. Animals had free access to water at all times. Similarly, males were kept in separate groups, except during the rut, during which some stags were brought into the females’ groups for mating.

On a weekly basis, deer were driven from the fields to a nearby handling facility, where they were weighed, their condition was monitored, and the number of bites on the pelt of each animal was classified as detailed in the previous sections. As a result, 40,159 animal monitoring events with information on pelt biting were achieved throughout the study (Table 2).

Animals were daily attended by qualified personnel, and an expert deer veterinarian (AJG) looked after the animals on a weekly basis. The farm complied with Spanish animal welfare legislation, and the monitoring procedure did not require an animal experimental license.

### 2.4. Hierarchy Rank

Between April and October of 2017 and 2018, agonistic interactions between 36 adult hinds (2017: group 1 = 9 deer, group 2 = 16 deer; 2018: group 1 = 16 deer, group 2 = 19 deer) were recorded using direct observations to estimate the linear hierarchy among animals.

Animal agonistic interactions (head butting, boxing, pelt biting, kicking, pushing, chasing, walking/running away, spatial displacement, visual threat) were recorded by one of the co-authors (ML-Q), with the aid of a pair of binoculars (8 × 42) and a telescope (20 × 60) from the top of a 4 m tall tower located at a vantage point in a plot where the hinds and their calves grazed. Observations took place between 08:00 h and 12:00 h, for a total of 521 h for 133 days, comprising 5067 animal interactions. Behavioural interactions were carried out by continuously scanning all animals (i.e., sampling as defined by Martin and Bateson [23]) and recording the type of behaviour, together with the identity of the pair of hinds involved and identifying which one was the aggressor and aggrieved. The frequency of interactions was generally low, which allowed the observer to record most of them, with some interactions missing during events of exceptional high activity.

To calculate the hierarchical rank of our animals, we used the Combi1 index (Equation (1)), together with the algorithm I&SI that minimises inconsistencies and ties in the calculation of the rank, implemented in Domicalc software [30]

Combi1 index = [*D_i_*/(*D_i_* + *S_i_*)] + *D_i_* − *S_i_*, (1)

where, *D_i_* and *S_i_* are the number of domination and subdomination events, respectively, of individual *i* over the rest of individuals in the group.

### 2.5. Heat Stress Index

We used meteorological data from the Spanish Ministry of Agriculture, Food, and Environment, supplied by SIAR regional service of Castilla-La Mancha (available at http://crea.uclm.es/siar/datmeteo/ (accessed on 21 April 2021) of July and August for the period 2006–2019. Data came from the meteorological station of Albacete (38°56′56.5′′ N 1°53′53.3′′ W), 2 km from the UCLM experimental deer farm and located at the same altitude. We used daily mean records across the study period of air temperature, relative humidity, wind speed, and global solar radiation to produce an index of heat stress [31,32] that has been used efficiently to assess thermal stress on red deer in outdoor conditions [13],

*THIWS* = 4.51 + 0.8 × *T* + 0.01 × *Hr* × (*T* − 14.4) + 46.4 − (1.992 × *W*) + 1.887 × *SR*, (2)

where *T* is the daily mean temperature (°C), *Hr* is the mean relative humidity (%), *W* is the mean wind speed (m∙s^−1^), and *SR* is the accumulated solar radiation over a 24 h circadian period (MJ∙m^−2^).

### 2.6. Statistical Analysis

As an exploratory hypotheses testing approach, we used GAM models (Generalised additive mixed models), implemented in the “*gam*” function of the mgcv R package [33]. The model showed that the GAM smooth relationships were in fact generally quite simple and could be well-described by simple polynomial functions. Consequently, we used linear mixed models with polynomial functions equivalent to those obtained by GAM models, implemented in the package lme4 [34] in R software version 3.4.1 [35]. For model selection, we used *p*-values against measures based on information theory, such as ΔAIC or BIC [36], as the objective was to identify the main drivers of the dependent variables. We proceeded by first fitting full models that included the explanatory variables and the pertinent interactions and then reducing the terms of the model using backward elimination by removing the non-significant fixed-effects interactions, one at a time, following the principle of marginality: the highest order interactions were tested first, and if they were significant, then the lower order effects were not tested for significance. Significance of the terms in the model was assessed using the R function lmerTest [37], which approximates degrees of freedom via Satterthwaite’s method, as in linear mixed-effects models, degrees of freedom are difficult to define appropriately [38]. The variance explained by the linear mixed model was represented as R^2^ marginal (variance accounted for by the fixed effects R^2^_LMM(m)_) and R^2^ conditional (variance accounted for by random and fixed effects; R^2^_LMM(c)_), following a method developed for linear mixed-effects models [39]. Calf ID, hind ID, and year were fitted as crossed random effects in the models. In order to not over-parameterise the models, we limited the number of interactions terms fitted. Graphics were constructed using ggplot2 R package based on the grammar of graphics [40].

## 3. Results

### 3.1. Pelt Biting Description

**Figure 2:**
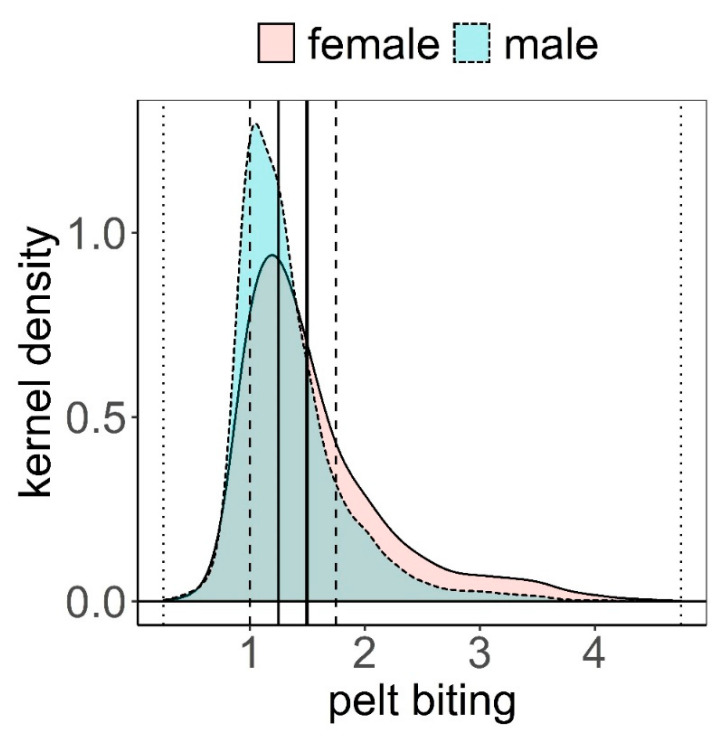
Kernel density estimation of pelt biting classes within female (f) and male (m) deer (see Methods for a definition of the classes). Mean (vertical solid thick line), median (vertical solid thin line), 1st and 3rd quartiles (vertical dashed lines), minimum and maximum (vertical dotted lines).

**Figure 3:**
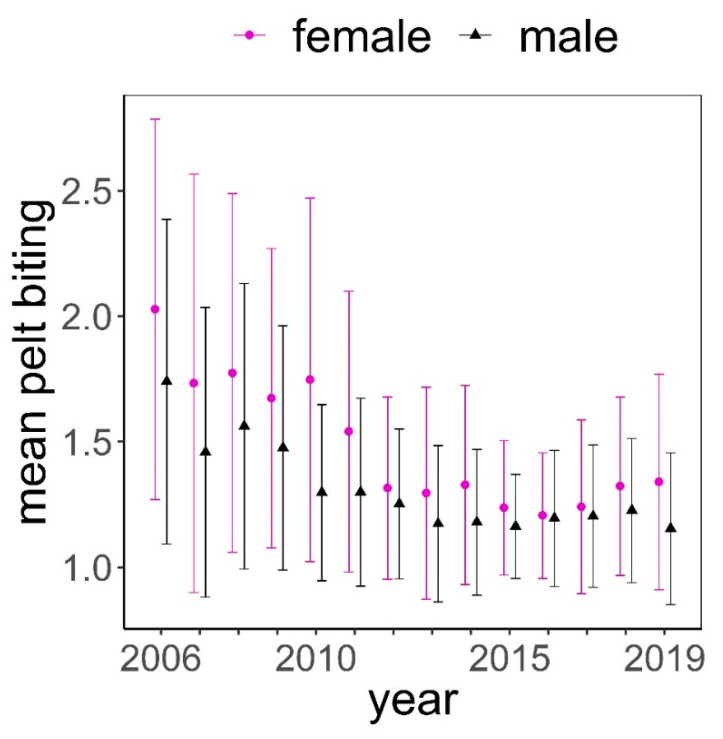
Mean (± standard deviation) of pelt biting classes in female (f) and male (m) deer throughout the study period.

The distribution of pelt biting classes was clearly biased towards the lowest classes (median = 1.25, mean = 1.50, Figure 2). The maximum class recorded was 4.75; this was exceptional, only recorded once in three different animals. These animals had no hair on most of their body, except head, lower parts of limbs, and underneath parts of the body. Frequency of pelt biting classes <1.5 was higher in males than in females, while it was more likely to find females exhibiting pelt biting classes >1.5 (Figure 2). Pelt biting was more intense for the period 2006–2011 compared to period 2012–2019, coinciding with a reduction in the number of deer in the farm that took place in 2011 (Figure 3).

### 3.2. Hypotheses Testing

**Figure 4:**
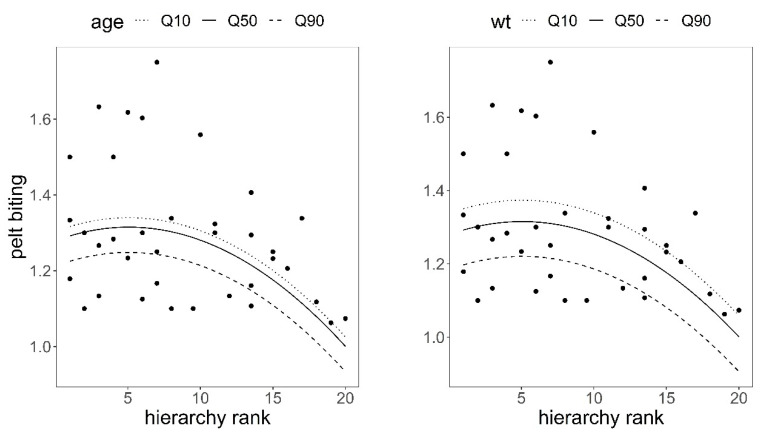
Predictions of the response of pelt biting to hierarchical rank in red deer hinds using model in Table 3. Variables not present in the plots were fixed to their means. Q10, Q50, and Q90 are 10, 50, and 90 quantiles. The higher the hierarchy rank, the higher the dominance.

There was a significant quadratic relationship between hierarchical rank and pelt biting in the observed group of 36 hinds; the higher the social rank, the lower the number of pelt bites after controlling for the effects of hind age and hind body weight (Table 3, Figure 4), which supported H1 (Table 1). Pelt biting responded to hind body weight and hind age, as predicted in hypotheses H2 and H3; the heavier and older the hind, the fewer bites on their coats, although the response was only significant for body weight (body weight coefficient = −0.005, SE = 0.0018, *p* = 0.009; age coefficient = −0.008, SE = 0.0044, *p* = 0.068, Table 3).

**Figure 5:**
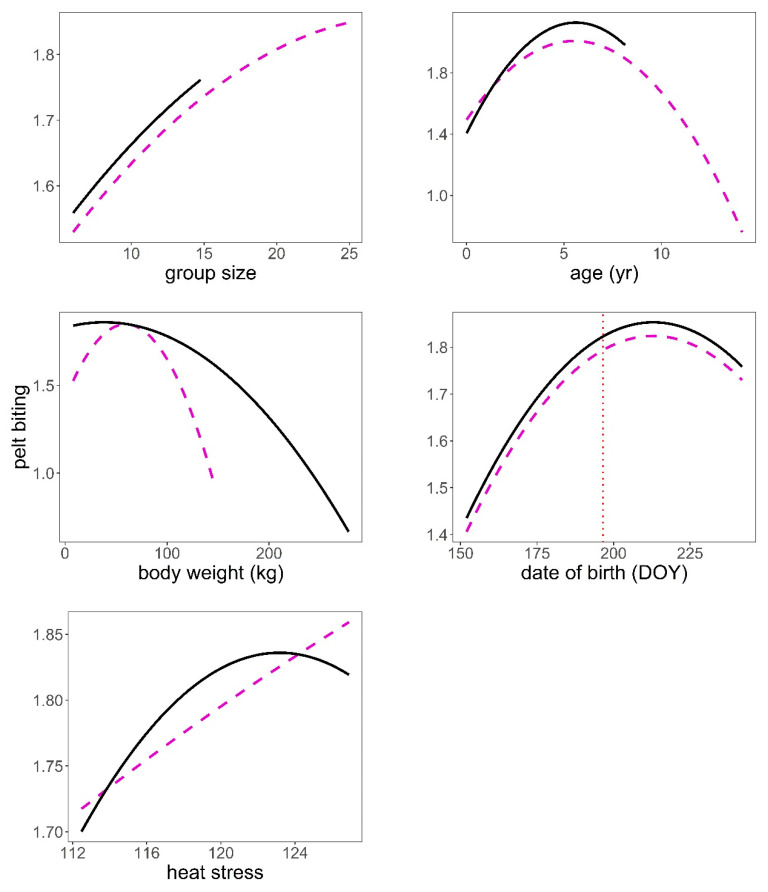
Predictions of the response of pelt biting to heat stress (THIWS, see Methods), group size, age (year), body weight (kg), and date of birth (DOY, day of year) based on model in Table 4. Male (black continuous line), female (magenta dashed line), mean date of births (vertical red dotted line). Variables not present in the plots were fixed to their means.

**Figure 6:**
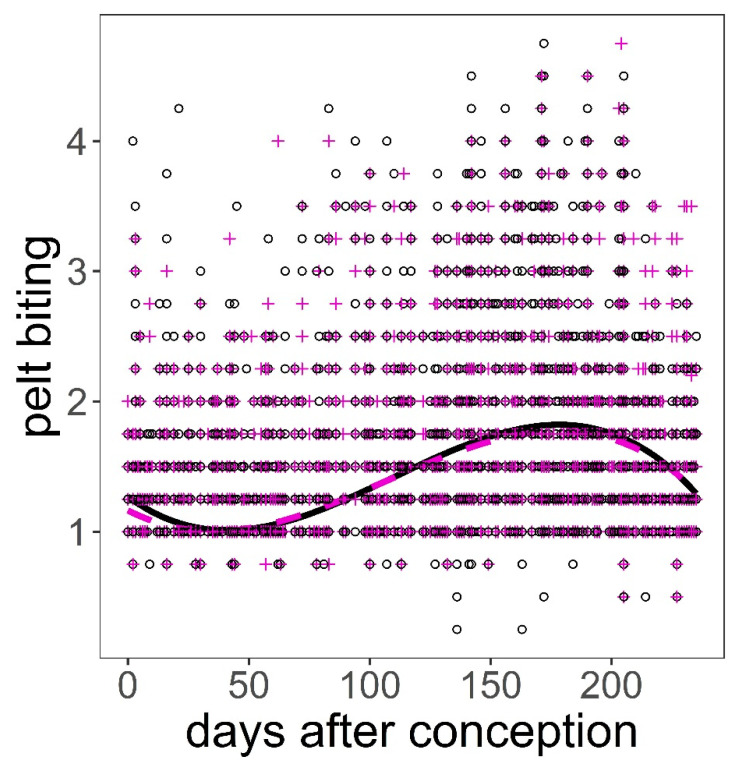
Predictions on the response of pelt biting on red deer hind to days after conception controlling for hind age and hind weight using model in Table 5. Black continuous line and black open circles are hinds carrying male foetuses; magenta dashed line and magenta crosses are hinds carrying female foetuses. Variables not present in the plots were fixed to their means.

The analysis of the pelt biting time series provided some support for hypotheses H2, H3, H4, and H6 after controlling for group size (Table 1, Table 4). The bigger the group, the larger the number of pelt bites exhibited on deer of both sexes (*p* < 0.001, Figure 5). We found a significant quadratic relationship between pelt biting and age in both sexes. The maximum number of pelt bites was observed in deer 5–6 years old; younger animals, but especially those in the oldest age classes, exhibited fewer pelt bites than 5–6 year-old deer (*p* < 0.001, Figure 5). The heavier the stag, the lower the number of bites on their coat; this pattern contrasted with that observed in hinds, which showed a peak in the number of pelt bites in hinds of average body weight, as compared to those lighter but especially to those heavier (*p* < 0.001, Figure 5). Hinds that gave birth earlier in the parturition period suffered less pelt biting than those that gave birth around the peak of the season (*p* < 0.001, Figure 5). It was clear that males and females suffered higher intensity of pelt biting as heat stress increased (H6) (*p* < 0.001, Figure 5). There was evidence that males suffered higher rates of pelt biting than females (H4) (male coefficient = 0.18, SE = 0.038, *p* < 0.001). Sackett et al.’s [27] hypothesis (H5) was not supported by our data; as a matter of fact, hinds carrying male foetuses were those that exhibited greater number of pelt bites than those carrying female foetuses, which was contrary to prediction (Table 5, Figure 6).

## 4. Discussion

The analyses clearly supported our hypotheses, except H5. On average, deer that were of higher hierarchical social rank (H1), heavier (H2), or older (H3) were bitten less by their peers than those that were of lower rank, lighter, or younger, respectively. Males were bitten more by same-sex peers than were females (H4), and biting behaviour increased in years of high heat stress (H6). All this being the case after controlling for group size, which had the expected significant effect, the larger the group, the higher the activity of pelt biting between its members. In addition, we found that hinds that gave birth earlier in the birth season suffered less biting than those giving birth at the peak of the season or later.

These results are relevant for deer farming, as they can be used to identify situations of social stress and put in place measures to minimise it in order to improve animal welfare and probably production. One of the most obvious measurements to control social stress in animal farms is by minimising spatial crowding [17,41]. Spatial restrictions can cause animal discomfort, even if they do not impose serious deprivations or injury [42]. Furthermore, appropriate spatial space facilitates natural social interactions that are beneficial to animal welfare [43]. Animal density is dynamic over time, as clustering level changes depending on the aggregation pattern of the animals [44,45]. For example, aggregation increases at feeding points in farms (feed bins, feedlots) but also in the wild, at points where food is scant and spatially concentrated (snow craters grazing in reindeer [4]). In an observational experiment carried out between 2017 and 2018, in the same facilities of this study and under similar population conditions, we observed that pelt biting rates were the same at the feeding belts as away from them (number of pelt biting observations at feeding belt = 350, away from feeding belt = 346, unpublished data). This suggests that, in our case, space restrictions impose conditions favourable to develop pelt biting behaviour. In contrast, in a nearby deer farm in which densities varied between 1.5–2.5 deer/ha and grazing was the main food resource, pelt biting was also observed, but it was an uncommon behaviour, as only a few individuals displayed bites on their pelt (Lagunes farm, Ciudad Real, Spain, unpublished data). In both farms, cases of extreme pelt biting were observed to be directed towards particular individuals of a group, and in one case, towards a hind and its 1-year-old calf, especially when individual animals were incorporated into an existing group. Similarly, in dairy goats, some individuals display consistent aggressive/submissive behaviour that identify them [46]. This points out that pelt biting can be useful to identify individuals that are being bullied and apply corrective measures consequently (e.g., move the bullied animal to a new group structure; identify and remove the bully animal from the group). In deer hunting ranches, where density reaches up to 0.4 deer/ha and food supplementation takes place when graze is scarce, pelt biting is infrequent, based on observations over four thousand legally culled red deer to which we had access to take samples for research purposes over several years [26,47], and we never found a case in which pelt biting exceeded class 1.7. This is expected, as free-ranging conditions hardly create favourable conditions for bullying; population density is generally low, sufficient space precludes oversized groups, and graze is widely dispersed and therefore difficult to defend and of low energy reward value per food item [48], although there are exceptions [4,22,49].

### Hierarchical Rank, Age, Body Weight, Date of Birth and Heat Stress

We predicted that animals with greater competitive abilities in agonistic encounters (those that were of higher hierarchical rank, heavier, and older) should receive less biting. Social rank has been found to be positively related to age and body weight in adult males and females of ungulate species [6,19,50,51,52]. On average, animals that are of higher hierarchical rank, heavier, or older are less likely to be challenged [53], and so are less bitten, than animals that are of lower rank, lighter, or younger. In contrast, animals at the bottom of the hierarchy might be aware of their limited competitive capabilities, and so respond by avoiding encounters that might result on being bitten when space is lacking. In a review, Miranda de la Lama and Mattiello [54] describe that goats attain their position in the social linear hierarchy at a very young age and tend to maintain this for a long time, independent of changes in the physical environment, although the introduction of new members to herds can increase aggression and, on occasion, alter the social hierarchy of the group. In goats, rank is very important for gaining access to resources; high-ranking goats gain access to more food than do low-ranking individuals [54].

Studies on wild red deer have shown that low-rank individuals leave their feeding station when peers of higher rank approach [55]; hinds that do not retaliate in fighting received less severe agonistic interactions than those that retaliate and lose [52]; in agonistic interactions between stags, 9% involved low-ranking individuals, 33% intermediate-ranking, and 58% top-ranking animals [26,52,56]. This could explain the quadratic effects between pelt biting and hierarchical rank, body weight, and age found in our hinds (Figure 4 and Figure 5). Namely, animals at the bottom of the hierarchy were bitten less, but those animals who had an average social rank, body weight, and age, therefore with some chances of winning, were keen to get involved in agonistic encounters to keep or increase their hierarchical rank, thus making them more likely to be bitten. This pattern was significantly more pronounced in females than in males for variable body weight, which could be explained by the fact that, in polygynous species, the reward of reproductive fitness is higher in males than in females [57]; thus, males are more prone than females to be involved in agonistic encounters and so be injured [26,52].

Caution is needed when assessing sexual differences in pelt biting in farmed deer, because they are probably affected by management. For example, in our setting, and in many deer farms, antlers are cut when they become hard to protect deer and staff personnel [58]. Males may have an average number of bites greater than females, because with no antlers, they are not able to antler fight and must resort to other agonistic behaviours, such as biting. On the other hand, males may have a lower number of bites than females due to receiving a more static management of social grouping than females. This is a consequence of males being kept in smaller and more stable groups, as their main use is mating in large harems [59]. On the other hand, female groups are more dynamic, as their management requires group reorganisation depending on breeding stage, age, and sales, which might lead to increasing agonistic behaviour to re-establish hierarchical relationships when group composition changes. This is supported by observations in wild red deer, where the number of escalated fights was greater when the animals meet unfamiliar conspecifics compared to the number of escalated interactions within same group members [52]. Furthermore, in reindeer, the reorganisation of groups led to increased fighting in order to establish a new hierarchy [60].

Our results on hypothesis H5 were contrary to the prediction [27]. It is not clear why our females carrying male foetuses suffered greater biting than those females with female foetuses, unless carrying one sex changes the aggressiveness of the mother, making her more likely to challenge higher-ranking mothers and thus suffer long-term retaliation. This result may be indirect evidence that deer can detect the sex of pregnant hinds, as was suggested in pigtail monkeys [27].

Heat stress increased the number of pelt bites in both sexes; this adds to further evidence that heat stress affects their behaviour and growth [13,61], and it should be taken into account as an important stressor, especially in farm conditions, when there are limited opportunities for heat abatement.

In seasonal breeders, such as red deer, timing calving to the period that concentrates vegetation growth is an advantageous strategy to meet the high nutritional demands of lactation and related reproductive fitness traits [62,63]. Births of male calves, the most costly sex, tend to be earlier than births of female calves (*Cervus dama* [64]; *Cervus elaphus* [51]). Early births in farmed red deer increases milk yield, milk energy, milk fat content, and calf growth and reduces body weight losses of hinds during lactation [65,66]. Early births are generally related with mothers being in good body condition [67]; this can be achieved by earlier exposure to spring green-up, being more efficient or competitive at grazing during the months before births, which might imply they were high rank animals that facilitated access for food resources [4,63,68]. We found that females that produced early births were less bitten than females giving birth later in the season; this suggests that they were high rank animals, which supports the findings of the previous studies commented on above.

## 5. Conclusions

Pelt biting responded to individual animal and social traits and could be useful as a conspicuous visual index to assess animal welfare in farm conditions. Because it is affected by a number of factors that make comparisons difficult between populations living under different conditions, it should be used as a monitoring tool to detect how social environment and management affect animal welfare within a population and to enable an according response with corrective measures. There is room to consider integrating pelt biting into automated image analysis systems, which are already being used to assess animal welfare, e.g., heat–cold stress, limping, and growth [69,70]. The value of our pelt biting index in wild populations is, however, limited, as pelt biting activity is expected to be low in the wild, but it should not be discarded as a diagnostic index in the animal welfare toolbox of game keepers and deer population managers.

## Figures and Tables

**Table 1:** Hypotheses and predictions on factors affecting pelt biting. Hypotheses are not mutually exclusive.

Group of Hypotheses	Terms	Hypotheses/Predictions
Life history traits related to hierarchy	Hierarchical rank	H1. Individuals of lower hierarchical rank suffer more pelt biting than individuals of higher hierarchical rank.
	Body weight	H2. Lighter individuals suffer more pelt biting than heavier individuals.
	Age	H3. Younger individuals are more likely to suffer pelt biting from older individuals.
	Sex	H4. Males are more likely to suffer pelt biting than females due to their higher intra-sexual interactions to achieve competitive skills for access to mating opportunities. H5. Pregnant hinds carrying female foetuses are more frequently attacked by conspecifics than those pregnant with sons, and so suffering more pelt biting.
Physical environment stress	Meteorological index of heat stress	H6. Heat stress conditions promote aggressions between animals within social units: animals exhibit more pelt biting during periods of high heat stress.

**Table 2:** Number of female and male red deer (n) and number of records across years (records).

	Year	2006	2007	2008	2009	2010	2011	2012	2013	2014	2015	2016	2017	2018	2019
females	n	137	178	193	205	158	126	79	29	38	40	51	61	61	56
	records	1800	3389	2915	3321	3245	2614	1665	709	874	1390	1188	1453	1613	1210
males	n	128	97	103	103	47	70	65	31	38	30	38	47	49	35
	records	1125	1470	1066	1002	846	1149	954	566	510	717	687	1035	1018	627

**Table 3:** Coefficients and statistics of a polynomial mixed model on the response of pelt biting to the hierarchical rank in red deer hinds, controlling for hind weight (body weight, kg), hind age (age, yr), hind identity (hind ID), and year. R^2^ marginal, variance accounted for by the fixed effects (R^2^_LMM(m)_); R^2^ conditional, variance accounted for by random and fixed effects (R^2^_LMM(c)_).

Random Effects	n	SD				
hind ID (intercept)	22	0.142				
year (intercept)	2	0.112				
residual		0.087				
no. observations	37					
Fixed effects	Coefficients of Polynomial Functions					
	Degree	Estimate	SE	df	*t*-Value	*p*
(intercept)	—	1.817	0.2361	14.7	7.696	<0.001
age	1	−0.008	0.0044	31.0	−1.888	0.068
body weight	1	−0.005	0.0018	31.1	−2.766	0.009
hierarchical rank	1	−0.495	0.1451	30.9	−3.409	0.002
hierarchical rank	2	−0.257	0.0918	30.5	−2.803	0.009
R^2^_LMM(m)_ = 0.436						
R^2^_LMM(c)_ = 0.848						

**Table 4:** Coefficients and statistics of a polynomial mixed model on the response of pelt biting to group size, age (in years), sex, date of birth (day of year), body weight (weight, kg), heat stress (THIWS, see Methods), animal identity (ID), and year. Female is the sex of reference. Other acronyms as in Table 3.

Random Effects	n	SD				
ID (intercept)	510	0.269				
year (intercept)	14	0.255				
residual		0.426				
no. observations	8154					
Fixed effects	Coefficients of Polynomial Functions					
	Degree	Estimate	SE	df	*t*-Value	*p*
(intercept)	—	1.35	0.073	15.8	18.68	<0.001
group size	1	8.36	0.981	5066.6	8.521	<0.001
group size	2	−3.22	0.653	7952.5	−4.926	<0.001
age	1	9.85	1.479	4840.4	6.664	<0.001
age	2	−11.85	0.894	8129.2	−13.259	<0.001
sex (male)	—	0.18	0.038	1315.1	4.897	<0.001
date of birth	1	8.49	0.492	7716.6	17.261	<0.001
date of birth	2	−6.37	0.541	7599.9	−11.773	<0.001
weight	1	−35.75	3.745	7901.0	−9.544	<0.001
weight	2	−35.64	2.735	8098.5	−13.033	<0.001
heat stress	1	3.84	0.698	7671.0	5.499	<0.001
heat stress	2	−0.12	0.632	7627.3	−0.185	0.853
age × sex (male)	1	4.30	3.966	7930.4	1.084	0.279
age × sex (male)	2	−4.34	3.628	8060.8	−1.196	0.232
weight × sex (male)	1	26.04	4.507	7623.1	5.777	<0.001
weight × sex (male)	2	29.54	2.892	8094.0	10.214	<0.001
heat stress × sex (male)	1	−0.91	0.925	7880.7	−0.986	0.324
heat stress × sex (male)	2	−1.73	0.907	7745.8	−1.908	0.056
R^2^_LMM(m)_ = 0.159						
R^2^_LMM(c)_ = 0.520						

**Table 5:** Coefficients and statistics of a polynomial mixed model to assess hypothesis H5 (in Table 1) on pelt biting. Female is the sex of reference. Other acronyms as in Table 3.

Random Effects	n	SD				
calf ID (intercept)	581	0.212				
hind ID (intercept)	156	0.325				
year (intercept)	14	0.253				
residual		0.430				
no. observations	8071					
Fixed Effects	Coefficients of Polynomial Functions					
	Degree	Estimate	SE	df	*t*-Value	*p*
(intercept)	—	1.49	0.076	16.0	19.658	<0.001
hind age	1	−2.00	2.313	246.9	−0.866	0.387
hind age	2	2.18	1.222	744.6	1.781	0.075
hind age	3	0.37	0.990	729.2	0.374	0.709
hind weight	1	−23.60	1.100	5656.9	−21.442	<0.001
hind weight	2	7.20	0.604	7770.5	11.921	<0.001
hind weight	3	−1.14	0.537	8032.4	−2.117	0.034
conception day	1	19.78	0.796	7811.5	24.854	<0.001
conception day	2	−8.56	0.663	7743.2	−12.907	<0.001
conception day	3	−12.08	0.644	7604.9	−18.753	<0.001
sex (male)	—	0.02	0.023	430.3	0.984	0.326
conception day × sex (male)	1	−0.18	0.885	7627.7	−0.207	0.836
conception day × sex (male)	2	0.56	0.875	7533.9	0.644	0.520
conception day × sex (male)	3	−2.33	0.870	7497.1	−2.679	0.007
R^2^_LMM(m)_ = 0.236						
R^2^_LMM(c)_ = 0.646						

## Data Availability

Data available on https://doi.org/10.5281/zenodo.5635977 (accessed on 1 November 2021).
